# Editorial: Artificial intelligence in forensic microbiology, volume II

**DOI:** 10.3389/fmicb.2024.1406563

**Published:** 2024-04-25

**Authors:** Chen Li, Yu-Dong Yao, Jiangwei Yan, Marcin Grzegorzek

**Affiliations:** ^1^Microscopic Image and Medical Image Analysis Group, College of Medicine and Biological Information Engineering, Northeastern University, Shenyang, China; ^2^Stevens Institute of Technology, Hoboken, NJ, United States; ^3^School of Forensic Medicine, Shanxi Medical University, Taiyuan, China; ^4^Institute of Medical Informatics, University of Lübeck, Lübeck, Germany; ^5^German Research Center for Artificial Intelligence, Lübeck, Germany

**Keywords:** artificial intelligence, pattern recognition, machine learning, forensic microbiology, medical information

Forensic microbiology is a branch of forensic science that involves examining various microbial characteristics to infer specific microbial sources and transmission pathways, thereby providing microbial evidence for legal purposes. In recent years, with the increased legal awareness among the population, there has been a rising proportion of civil litigation cases involving passive infection. The scope of forensic microbiology identification has gradually expanded from tracing microbial sources in the environment and hosts to identifying the spread of pathogens between individuals and assessing the damage caused by such transmission. As human cytomegalovirus is a common and infectious microorganism with humans as the primary host, it has become a subject of forensic microbiology. While in most cases, human cytomegalovirus infection does not manifest obvious symptoms, a considerable portion of patients with compromised immune systems may experience acute symptoms, including sepsis and neurological infections, after infection or post-infection, along with potential long-term tumor risks. There is an association between human cytomegalovirus and glioblastoma, with human cytomegalovirus infection considered to increase the risk of glioblastoma, and detection of human cytomegalovirus in glioblastomas. At the same time, the presence of epilepsy symptoms is also one of the crucial criteria for identifying glioblastoma-related damage.

In a study led by Wang et al., a total of 142 patients from two centers were included. All patients underwent MRI examinations and were pathologically diagnosed with glioblastoma. The data for the training and validation sets were from the same center, while the data for the test set came from another center. Patients were divided into two groups based on whether they experienced epilepsy. The researchers employed various deep learning methods to predict the occurrence of non-surgically related epilepsy in glioblastoma patients and compared the results with models established using machine learning methods. The best model achieved an accuracy of 73.3%, demonstrating the potential of deep learning in this context and providing a basis for the identification of injuries related to human cytomegalovirus (Wang et al.).

Meanwhile, Yao and Zhang's review discussed the forensic applications of machine learning in Human Papillomavirus (HPV) detection. Xu et al.'s review discussed the application of machine learning in forensic bacterial identification, particularly concerning issues of identity, race, and the location of cases. Wu et al.'s review discussed the use of artificial intelligence to analyze microbiome data for assessing postmortem interval and crime scene location.

With the continuous development of detection technologies and changes in the types of litigation, the application scope of forensic microbiology is gradually expanding. Microbial identification goes beyond classification, encompassing the sources of microbial transmission and the sequence of pathogen infections. Such as, through machine learning methods, it is possible to uncover the complex relationship between the mutation of the virus during replication and the changes in its disease-causing ability. However, microbial communities are influenced by various factors, and their genetic information is prone to mutations during the transmission process. This reminds researchers to compare their results with publicly available databases to improve robustness. This prompts collaboration between forensic researchers and experts from other fields to establish more in-depth feature extraction methods.

## Author contributions

CL: Conceptualization, Funding acquisition, Project administration, Writing – original draft, Writing – review & editing. Y-DY: Conceptualization, Writing – review & editing. JY: Conceptualization, Writing – review & editing. MG: Conceptualization, Writing – review & editing.

